# Supplementary material for: A Survey on the Evaluation of Monosodium Glutamate (MSG) Taste in Austria
Source: Foods. 2024 Dec 25;14(1):22. doi: 10.3390/foods14010022 (PMC11719957; doi:10.3390/foods14010022)
Supplement: Supplementary file 1 [file foods-14-00022-s001.zip › foods-3357330-supplementary.pdf]

Supplementary Materials:

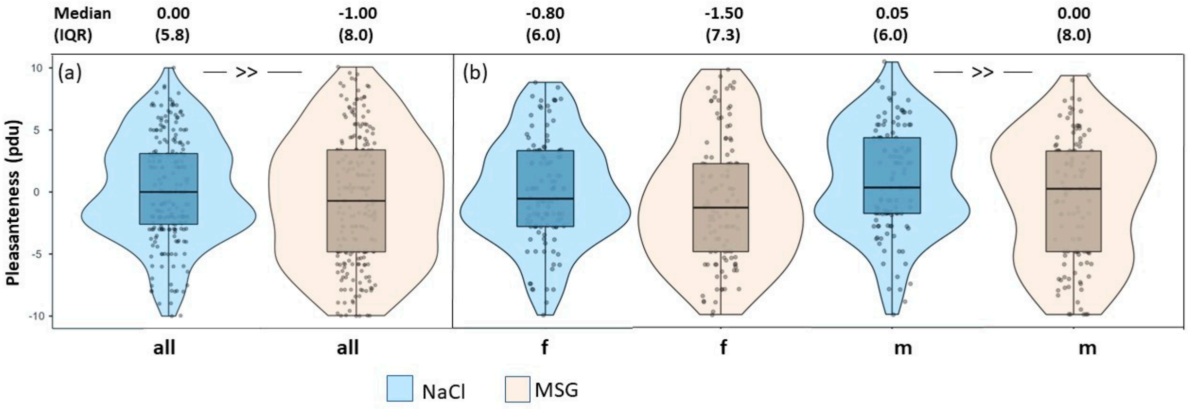

SM-Figure1 Violin plots of pleasantness with overlaid box plots based on median and interquartile range (IQR) describing the ratings for salt (NaCl) and umami (MSG) obtained from the entire group (a) and from the gender subgroups (b). The overall sample and the male subset rated the MSG solution less pleasant than the NaCl. The statistical significance levels of the results are highlighted with “>” where the opening of the angle bracket is in the direction of the greater value. Also: “>>” indicates p<.01.
